# Mechanochemistry as an emerging tool for molecular synthesis: what can it offer?

**DOI:** 10.1039/c7sc05371a

**Published:** 2018-03-07

**Authors:** Joseph L. Howard, Qun Cao, Duncan L. Browne

**Affiliations:** a School of Chemistry , Cardiff University , Main Building, Park Place , Cardiff , CF10 3AT , UK . Email: dlbrowne@cardiff.ac.uk

## Abstract

Mechanochemistry is becoming more widespread as a technique for molecular synthesis with new mechanochemical reactions being discovered at increasing frequency. Whilst mechanochemical methods are solvent free and can therefore lead to improved sustainability metrics, it is more likely that the significant differences between reaction outcomes, reaction selectivities and reduced reaction times will make it a technique of interest to synthetic chemists. Herein, we provide an overview of mechanochemistry reaction examples, with ‘direct’ comparators to solvent based reactions, which collectively seemingly show that solid state grinding can lead to reduced reaction times, different reaction outcomes in product selectivity and in some instances different reaction products, including products not accessible in solution.

## Abstract 2

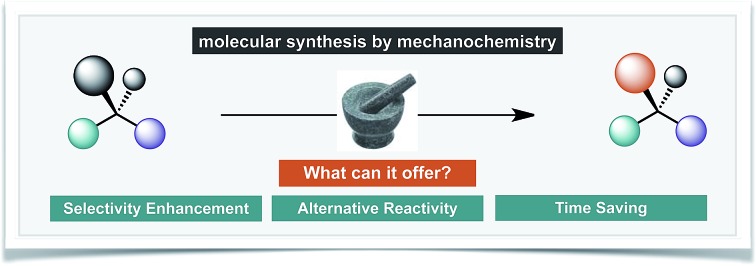
Mechanochemistry is becoming more widespread as a technique for molecular synthesis with new mechanochemical reactions being discovered at increasing frequency. This perspective explores what more it can offer, aside from the clear benefit of reduced solvent consumption.

## 1. Introduction

Mechanochemistry, well known in the area of crystal engineering and polymorphism, is re-emerging as a technique for organic synthesis.^1^,^2^ Whilst the technique is not new, it is now evolving beyond simply being a solvent free alternative. Despite many synthetic transformations now reported, perhaps the most interesting transformations are those that exhibit different reactivity to conventional solution-based reactions.^3^,^4^ In this perspective, we highlight a selection of such examples as well as provide a brief overview and introduction to the topic.

### 1.1 What is mechanochemistry?

A mechanochemical reaction is defined as “a chemical reaction that is induced by the direct absorption of mechanical energy”.^5^ Mechanochemistry therefore complements the conventional methods of activation: heat, irradiation and electrochemistry.^6^ It is also related to tribochemistry, in which chemical reactions occur on the surface/boundary between different environments.^7^,^8^ Despite a recent resurgence in mechanochemistry, it remains far less well studied and understood in comparison to conventional methods of energy input.

It is well understood that chemical reactions, initiated through different forms of energy input, can lead to different products and provide access to reaction manifolds that are inaccessible by other means; *e.g.* photochemistry and electrochemistry. For example, pericyclic reactions proceed differently depending on whether they are induced by light or heat. The reaction outcome is predictable through use of the Woodward–Hoffmann rules.^9^,^10^ Interestingly, when ultrasound is used as the input energy for a pericyclic reaction, products have been observed that are not predicted; indeed it has also been suggested that Woodward–Hoffmann rules should not necessarily be applied to mechanochemical pericyclic reactions.^11^,^12^

Mechanochemistry therefore presents an opportunity to explore a novel chemical space of reactions. Mechanochemistry can describe impact or pulling forces.^13^ This perspective focuses on impact caused by milling.^14^–^19^

### 1.2 What are the types of milling device?

The earliest mechanochemical reactions were carried out using a pestle and mortar.^20^ However, how these reactions behave is highly operator dependent, as each individual may impart different levels of energy. Running reactions for longer than a few minutes also becomes challenging, and depends on the operator's stamina! Therefore, electronic milling devices are commonly used.

**Scheme 1:**
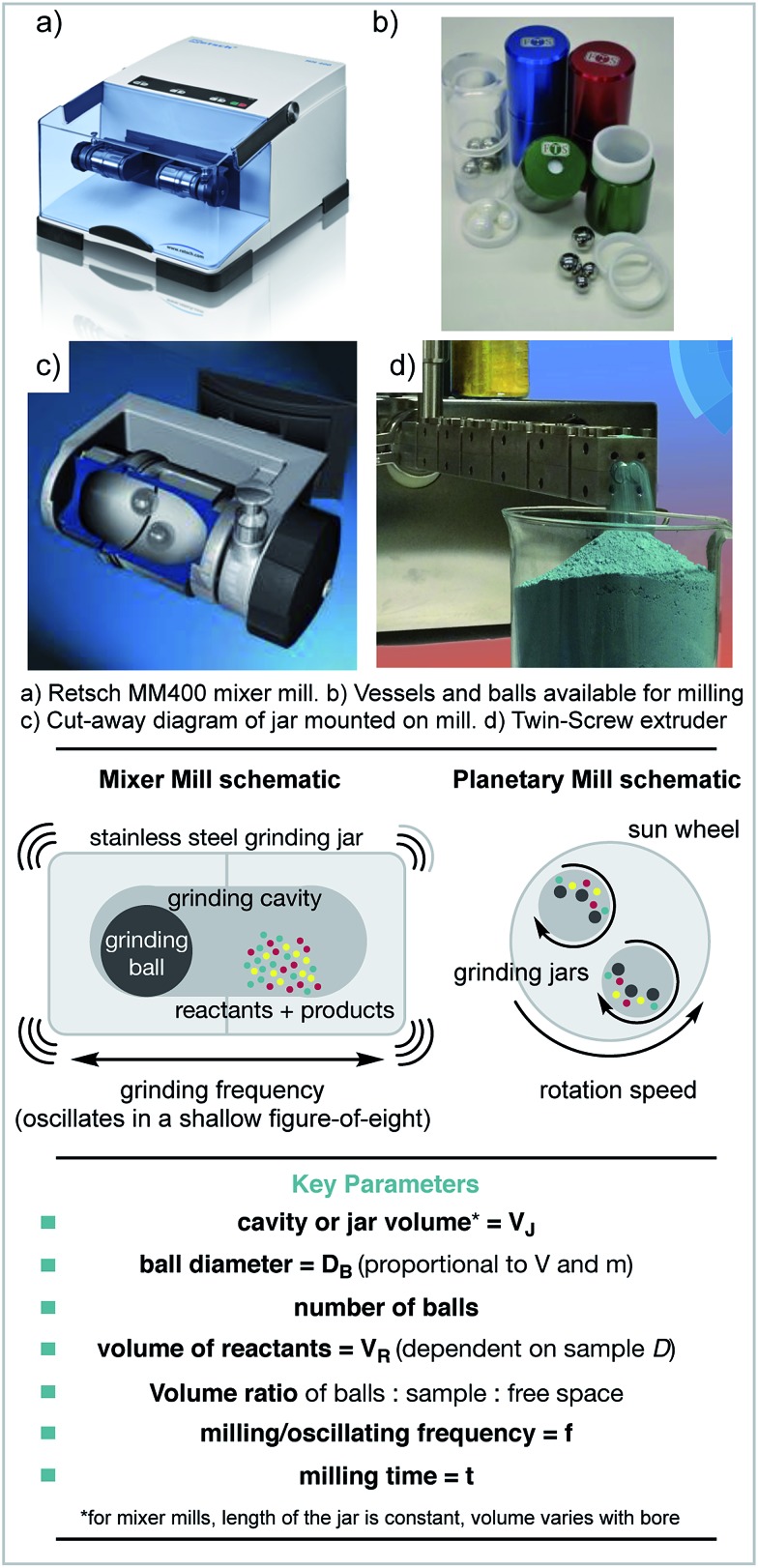
Examples of equipment used for mechanochemical reactions and key parameters. (a & c) Reproduced with permission from Retsch, (b) reproduced with permission from FormTech Scientific. (d) Reproduced with permission from Prof Stuart James and RSC.

The mixer mill is one type of ball milling machine, which uses the movement of ball bearings to apply mechanical force to the reagents. In this case, reagents are loaded into jars and one or more ball bearings are added. The jars are then mounted horizontally and shaken at the desired frequency (Scheme 1). The main mechanical energy applied to the reagents is impact force. The other most common type of ball mill is a planetary mill. In this case, the reagents and ball(s) are loaded as before, but the motion is different. In this instance, the jars spin counter-directionally to the spinning disc that they are mounted on. The central spinning disc is called the ‘sun wheel’ and the jars orbit around the central spinning axis. In both cases, the material and size of the jars and balls can be altered. The main type of force applied in planetary milling is shear force. Stirred media ball mills are also available, in which the reagents and balls are stirred together. This type of mill is commonly used to create very fine powders, *e.g.* of coal.^21^,^22^ Stirred media reactions or slurries can also be set up in round bottom flasks or more traditional lab glassware.^23^

### 1.3 What scales?

If any synthetic procedure is to be useful to society *e.g.* in molecule discovery or manufacture it must be achievable across a variety of scales. For mechanochemistry, each type of milling device can achieve different scales. The mixer mill can achieve gram scales, which are suitable for lab investigations. However, for anything larger, other types of mill must be used, and different sizes of planetary mill are available. However, for pilot and manufacture scales, stirred media ball mills are used. For example, the Outotec HIGMill has a volume of 30 000 litres and can be used at >1000 kg scales.^24^

Stolle *et al.* demonstrated the scalability of the mechanochemical Knoevenagel condensation between vanillin and barbituric acid in a planetary mill, scaling from 20 to 300 mmol.^25^ This resulted in the ability to obtain quantitative yields on approximately 80 g scales within short reaction times.

A different approach to scalability can be attempted if the process is modified from batch to continuous. This can be achieved by using extruders instead of mills. Extruders continuously force material through confined spaces and apply shear and compression forces. James, Crawford and co-workers have synthesised MOFs at rates of kilograms per hour by making use of twin-screw extruders.^26^

### 1.4 What are the variables?

As when performing any reaction, understanding and controlling the variables is important. However, when using a ball mill it can be challenging to control or even measure the variables individually. There are three main variables that affect how mechanochemical reactions perform: the kinetic energy of the ball(s) prior to collision, how that energy is transferred to the reagents and the frequency of collisions.

The amount of kinetic energy that the ball(s) possess prior to collision is the maximum amount of energy that can be transferred to the reagents per collision.

In a collision, how that energy is transferred can have an effect on whether or how a reaction occurs. This can be by direct impact, under which the material is locally compressed, or by shear force in which a reactive face is exposed. It has been shown that these different types of energy absorption can lead to different outcomes.^27^ Different types of ball mill achieve different ratios of impact and shear forces.

Differences in mixing or mass transfer, can affect the outcome of any reaction. In solution, this is easily controlled by stirring, mass transfer is rarely a problem on small scale. However, in milled reactions, this can be a difficult variable to control and may have a dramatic effect on the outcome of a reaction. To help homogeneity and mixing, grinding agents can be used (see Section 1.6).

Finally, those variables that are usually changed in any standard reaction optimisation also apply, such as stoichiometry, reaction time and temperature. However, temperature is not a parameter that is easily controlled, the reaction vessel heats up due to the collisions and has a dependence on the filling degree volumes of grinding balls, sample and vessel size as well as oscillating frequency. Cryo-mills are available, which consist of pausing the grinding process to automatically cool the jars with liquid nitrogen but are not widely explored for chemistry purposes.^28^

The previous section discussed the variables that affect mechanochemical reactions. However, these variables cannot be directly controlled. The variables that can be controlled often have multiple influences on a reaction. One of the first variables to be decided is what type of ball mill is to be used. As discussed previously, there are three main types: the mixer mill, the planetary mill and the stirred media mill. It has also been demonstrated that different mills can lead to differences in reaction performance.^29^–^32^

The next consideration is usually given to the filling degree. This is a measure of how full the jars are, considering the volumes occupied by the reagents and balls compared to the total cavity volume of the jars. This has a significant effect on the trajectories of the balls, and therefore the energy transfer and mixing. The effect of the milling ball filling degree has been investigated for the Knoevenagel condensation of vanillin with barbituric acid in a planetary mill.^23^ For this specific example it was observed that having the balls filling approximately 25% of the total volume was optimal. However, this may not be the case for other reactions and/or other types of mills.

The milling frequency is perhaps the easiest variable to control and can be changed simply by adjusting the settings on the mill. When increased, this increases the velocities of the balls, and so increases their kinetic energy. This is a facile way to change the energy input into a reaction.

### 1.5 Which reactions are best suited to mechanochemistry?

As so many of the mechanisms operating mechanochemically remain elusive, it can be difficult to decide when to use mechanochemistry. Certainly, many of the examples presented here, which exhibit different reactivity in solution, are unlikely to have been predicted. However, there are a few cases where definite advantages over solution-based reactions can be expected *a priori*.

Perhaps the most obvious advantage is that reactions can be performed under solvent-free conditions. This leads to several cases where it is worth attempting mechanochemical reactions.

Firstly, reactions between solids that are not soluble (or not all components are soluble in the same solvent) are well suited to mechanochemistry. This class of reactions can be very challenging, or even impossible in solution. Reactions in which the solvent can interfere are also interesting candidates for mechanochemical investigations. For example, many catalysts and reagents can be very sensitive towards water, or solvents with Lewis basic sites. Indeed, great lengths are often taken, with expense, to dry solvents. However, in the mill the solvent is not required. Finally, reactions that require hazardous solvents could be made safer by using solvent-free conditions, such as mechanochemistry.

So far, only reactions between solid reagents have been discussed, although reactions between solids and liquids, or even liquids can be performed mechanochemically. These usually require the addition of a solid material referred to as a grinding agent or grinding auxiliary.

### 1.6 What is a grinding auxiliary/agent?

Reactions in the mill that feature one or more liquid component (starting material, reagent or product) often require the addition of a solid additive for efficient reactivity. It has been shown that different textures of reaction mixtures can lead to different reaction kinetics.^33^ When liquids are used, the reaction mixture can become sticky and resemble a paste or gum, this prevents efficient mass and energy transfer. To account for this, it is common to use an auxiliary material such as silica, alumina, talc or inorganic salts as a milling agent/auxiliary or adsorbent.^28^,^34^ In pharmaceutical formulation science these materials are termed ‘glidants’ or ‘lubricants’ and assist in the uniform passage of powdered materials through screw extruders.^35^

It is important that such additives are chemically inert towards the desired reaction, otherwise they might interfere with the reactivity; proving inertness is difficult!

### 1.7 What is liquid-assisted-grinding (LAG)?

The mechanochemical reaction environment can be modified further by the addition of a small amount of liquid. This is termed “Liquid Assisted Grinding” (LAG). The amount of added liquid has been characterised by the parameter *η*, which is a ratio of the volume of liquid added to the total mass of reagents.^36^ This ratio can vary from grinding neat reagents together with no liquid, through to LAG, slurry reactions and eventually to solution reactions with increasing amounts of liquid.

LAG was originally used in mechanochemical cocrystallisation and it was found to speed up cocrystal formation.^37^ It has also been demonstrated that LAG can lead to different results to slurry reactions and that the outcomes do not necessarily depend on the solubility of the starting materials in the liquid used.^34^ More recently, it has been shown that using different amounts and types of liquid can lead to the formation of different polymorphs, dispelling the commonly held belief that polymorphism depends solely on the solvent.^38^

These observations show that LAG can lead to different and currently unpredictable reaction outcomes compared to both neat grinding and solution based processes. It is therefore an added variable when exploring solid state grinding.

### 1.8 Expect the unexpected

Given the significant difference in reaction environment between conventional methods and mechanochemical methods, it is reasonable to suggest that one would expect to observe significant differences in reaction outcomes. Given the many mechanisms and processes involved, what these differences might be is not easily predicted. When one takes into account the interdependent nature of the variables in a mechanochemical reaction, and further modifications, such as LAG, it becomes even more complex and challenging to know what to expect. As such, it is likely that in many of the examples presented in this perspective, the outcomes were unexpected. However, we have attempted to combine many of the recent examples and classify them into three categories: time saving (in the form of reduced reaction time or greatly enhanced yield over a similar reaction time), selectivity enhancement and alternative reactivity. To highlight the differences these have been compared to solvent-based reactions; where these are known. This overview is to raise awareness or the capabilities of the technique and enable others to explore this exciting method rather than to necessarily advocate the use of mechanochemistry instead of solution based approaches.

## 2. Reduced reaction times

One way in which mechanochemistry offers apparent advantages over solution based reactions is a reduction in reaction times. Clearly this in part derives from a large increase in concentration. It may also be true that in some cases there is a temperature difference between the solution based reaction and the mechanochemical process. In general the control of instantaneous temperatures under milling conditions is difficult to achieve, and control over the bulk temperature relies on bespoke equipment. Nonetheless there are many reactions where the comparison to running a solvent based reaction to an ‘ambient’ mechanochemical reaction demonstrate significant reductions in reaction time and we highlight some of those examples in this section.

**Scheme 2:**
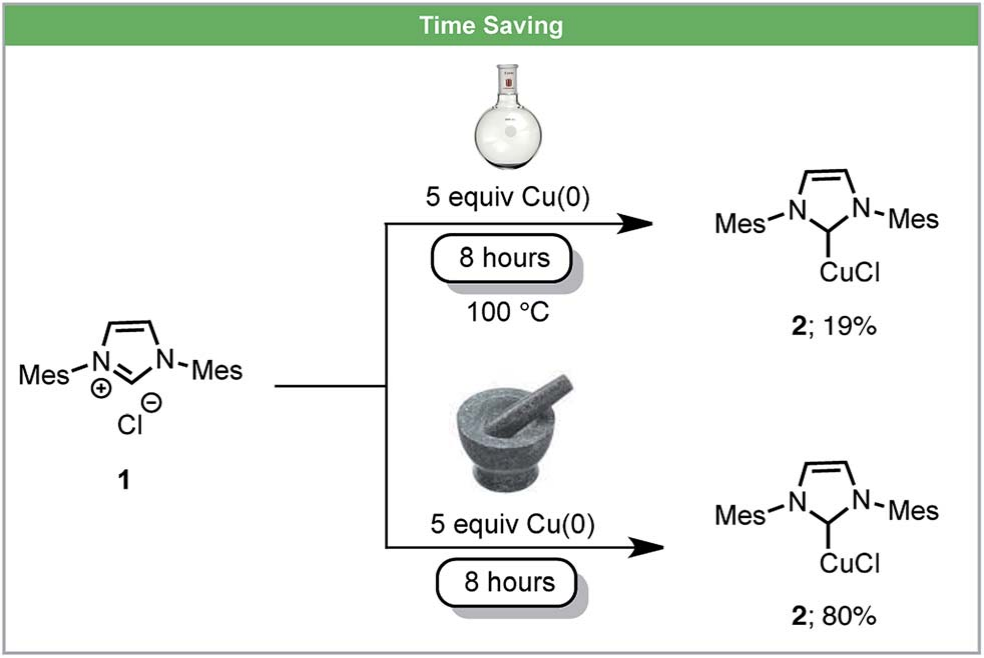
Formation of Cu–NHC complexes; Lamaty and co-workers.^41^

An example of an inorganic reaction with increased reactivity is the synthesis of Cu–NHC complexes (NHC = N-heterocyclic carbene). These are used widely as organometallic catalysts for a variety of reactions and various methods have been developed for the synthesis of Cu–NHC complexes.^39^ Under solvent-based conditions, Cu–NHC complexes can be synthesized by the reaction of metallic Cu(0) with imidazolium salts, although these reactions require a large excess of insoluble Cu(0) and long reaction times.^40^ Recently, Lamaty and co-workers reported that Cu–NHC complexes (**2**) could be synthesized from imidazolium salts (**1**) and metallic copper using a planetary ball mill (Scheme 2).^41^ The rate of reactions was enhanced due to the high concentration of reagents and the highly efficient mixing under mechanochemical conditions. Using this novel method, five Cu–NHC complexes with different counter ions (Cl^–^, BF_4_^–^, and PF_6_^–^) were successfully synthesized in improved yields compared to the analogous reactions in solution.

One of the most powerful tools for chemical synthesis is the selective functionalization of C–H bonds. Such methods allow the formation of C–C bonds without prefunctionalisation of the starting materials.^42^ There are already several known methods for C–H activation and functionalisation using mechanochemical conditions, some of which provide a time saving against the analogous reactions in solution.^43^

**Scheme 3:**
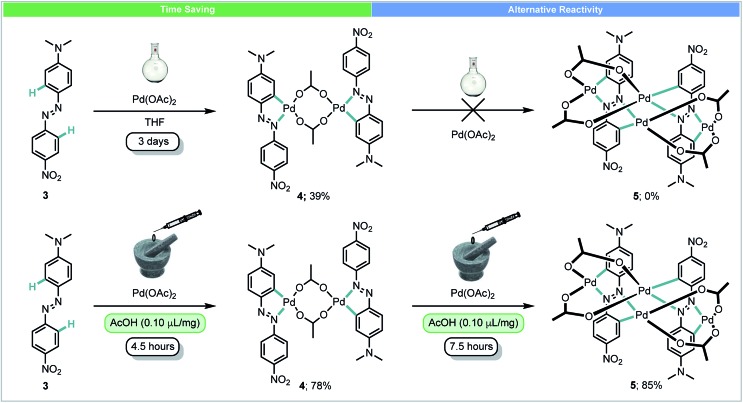
Palladacycle synthesis; Ćurić and co-workers.^44^

In 2014, Ćurić and co-workers achieved the first mechanochemical transition-metal-mediated C–H bond activation and monitored the transformation with *in situ* solid-state Raman spectroscopy.^44^ Using liquid-assisted grinding (LAG) with acetic acid, palladacycle **4** was synthesized from asymmetrically substituted azobenzene **3** and Pd(OAc)_2_ in 78% yield after 4.5 hours (Scheme 3). When performed in solution, this reaction required 3 days and a significantly poorer yield was achieved. Further milling of **4** with Pd(OAc)_2_ yielded dicyclopalladated complex **5**, which was not observed after multiple attempts at the same transformation in solution. In addition to saving time, this example demonstrates that using mechanochemistry can offer novel reaction pathways for the synthesis of organometallic compounds which could not be obtained using other methods.

**Scheme 4:**
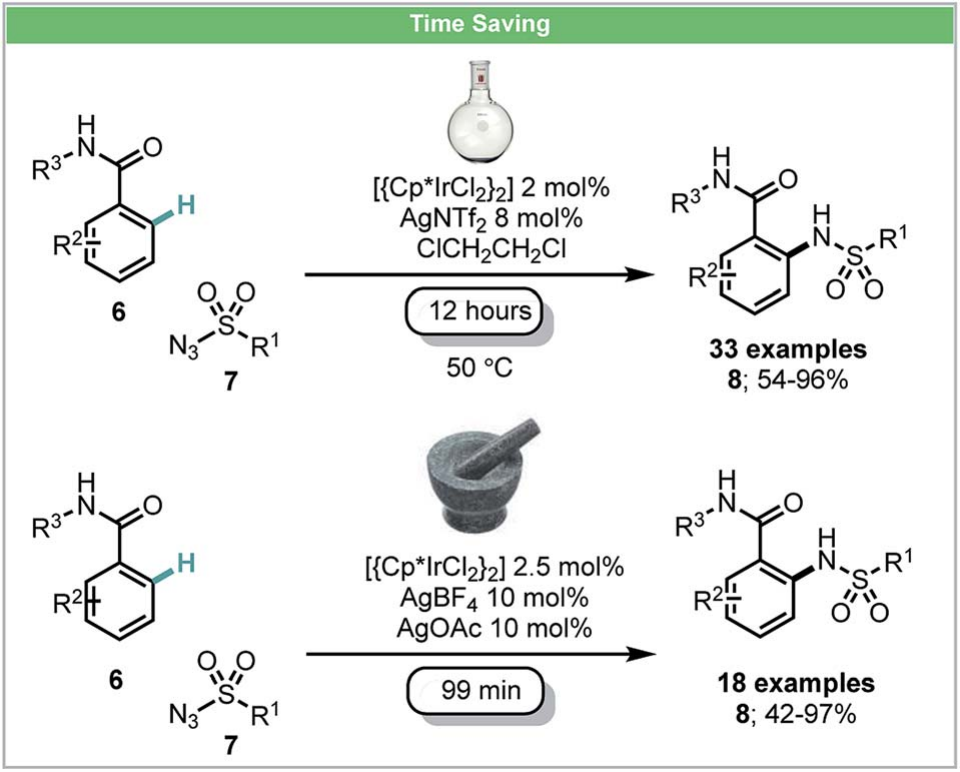
C–H amidation of benzamides; Bolm and co-workers.^45^

In 2016, Bolm and co-workers developed a mechanochemical iridium(iii)-catalyzed C–H bond amidation of benzamides **6** with sulfonyl azides **7** (Scheme 4).^45^ In this study, it was demonstrated that the active cationic Ir(iii) catalyst could be formed *in situ* in the mixer mill by reaction of [{Cp*IrCl_2_}_2_] with AgNTf_2_. The corresponding amidated products could be obtained in high yields with shorter reaction times (99 min) than those under a solvent-based protocol (12 hours) as reported by Chang and co-workers.^46^

**Scheme 5:**
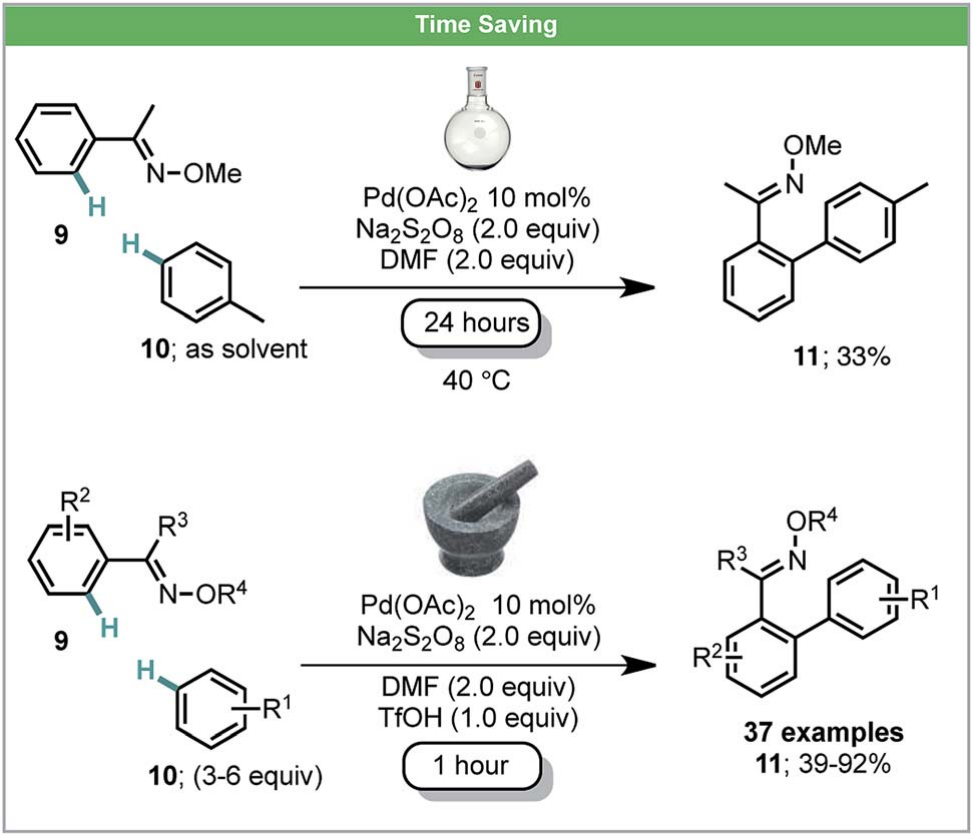
Oxidative C–H/C–H coupling; Xu and co-workers.^48^

Oxidative C–H/C–H coupling has the potential to become a very powerful tool for sustainable chemical synthesis as no starting material prefunctionalization is required for either coupling partner.^47^ Ball-milling has also been used for dehydro-C–C coupling reactions with an illustrative example having been developed by Xu and co-workers.^48^ Under mechanochemical conditions, biaryl products **11** could be obtained in both high selectivity and yield within a one hour reaction time. Specifically, electron-deficient oximes **9** were treated with a variety of arenes **10** in the presence of a palladium catalyst and oxidant (Scheme 5). Anilides were also found to be competent directing groups for this transformation. The comparable reaction in solution, after stirring for 24 hours using toluene as both a solvent and reagent, achieved a poorer yield than any mechanochemical results. Similar solution based methods for coupling anilides with arenes developed by Yu,49*a* Dong,49*b* and You49*c* also usually require more than 16 hours for the reaction to be complete. By employing ball milling, only 3–6 equiv. of simple arenes were required. It is also worth noting that electron-deficient arenes such as acetophenone and fluorobenzene were found reactive using this method, which were less studied in other C–H/C–H reports.^47^

**Scheme 6:**
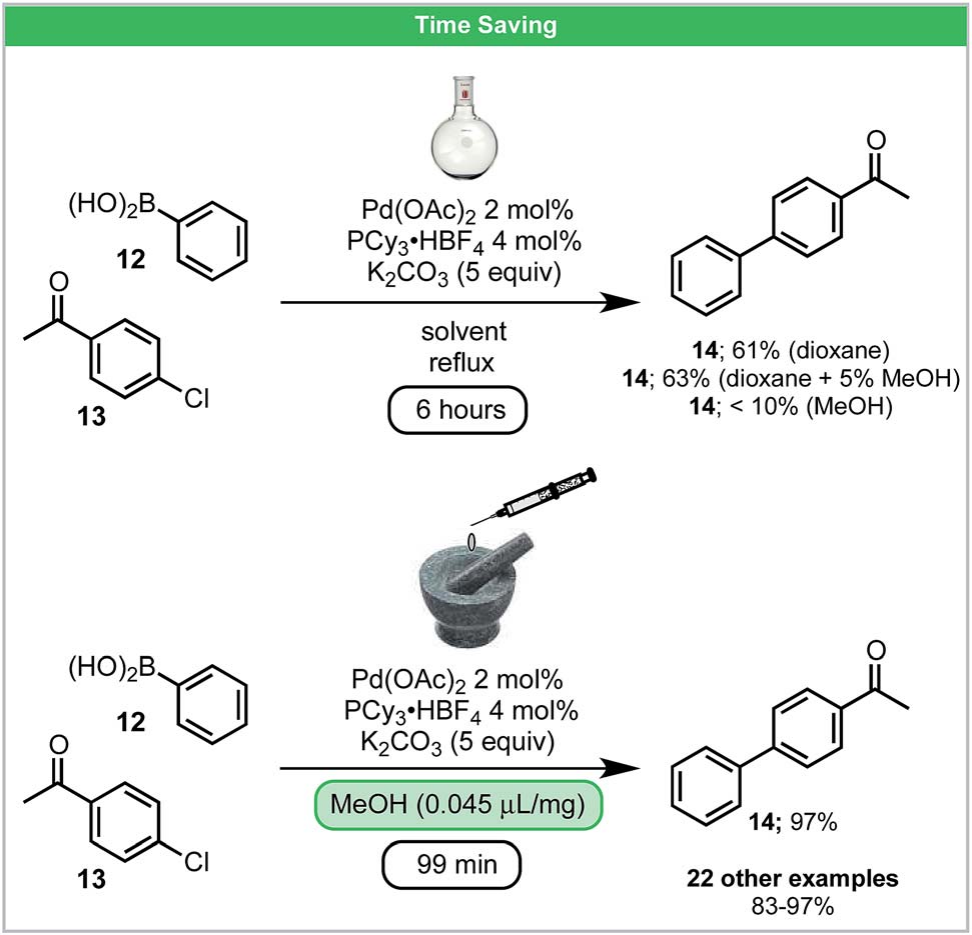
Suzuki–Miyaura cross coupling; Su and co-workers.^50^

In 2016, Su and co-workers developed a LAG accelerated, palladium catalysed Suzuki–Miyaura coupling of aryl chlorides **13** with boronic acids **12** under ball-milling conditions (Scheme 6).^50^ Compared to the solvent-based reaction (Scheme 6), higher yields in shorter reaction times could be achieved. Adding solvents, which are commonly used in Suzuki–Miyaura reactions (THF, dioxane, DMF or MeCN), as LAG agents, did not lead to improved results.^51^ However, protic solvents such as alcohols/H_2_O led to improved reactivity. It was proposed that under these conditions alcohols form alkoxides *in situ*, which could participate in both ligand exchange and boronic acid activation. This may explain the improved reactivity observed using LAG. In addition, it was also shown that much lower catalyst loading, 0.5 mol% Pd with 2.5 equiv. K_2_CO_3_, could be used when the reaction was scaled up to gram scale.

**Scheme 7:**
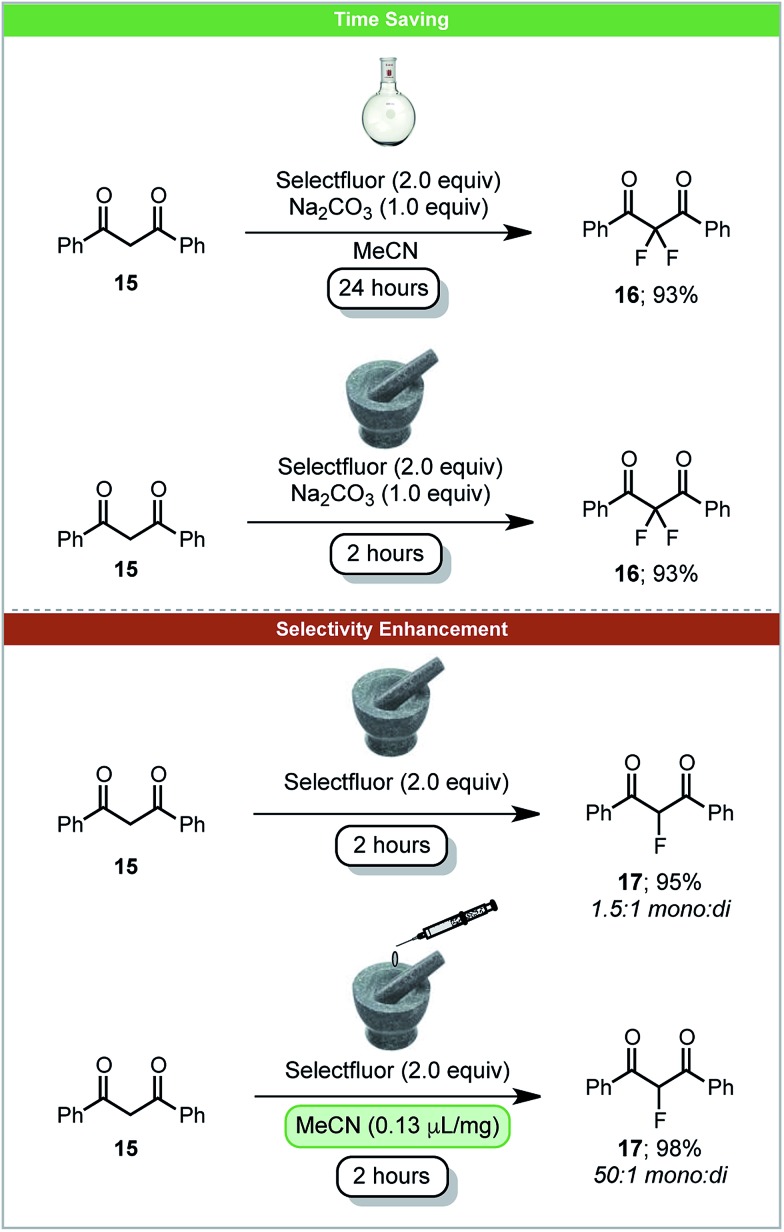
Fluorination of 1,3-diketones; Browne and co-workers.^52^

The carbon–fluorine bond is present in a wide range of valuable chemical products, such as pharmaceuticals and agrochemicals. In 2017 the first carbon–fluorine bond formation under solid-state mechanochemical milling conditions was reported. Simple C–F bond formation was achieved by fluorinating 1,3-diketones **15** using Selectfluor as an electrophilic source of fluorine (Scheme 7).^52^ It was observed that by using LAG with acetonitrile, the selectivity of monofluorinated products **17** was enhanced in comparison to neat grinding. This demonstrates the significant effects LAG can have on a reaction's outcome. Difluorination could be achieved by the addition of sodium carbonate under solvent free conditions within two hours. The analogous solvent based reaction requires 24 hours (Scheme 7). When using the less reactive β-keto ester, solvent based reactions take five days to complete, whereas under ball-milling conditions reaction is complete within two hours.^53^

**Scheme 8:**
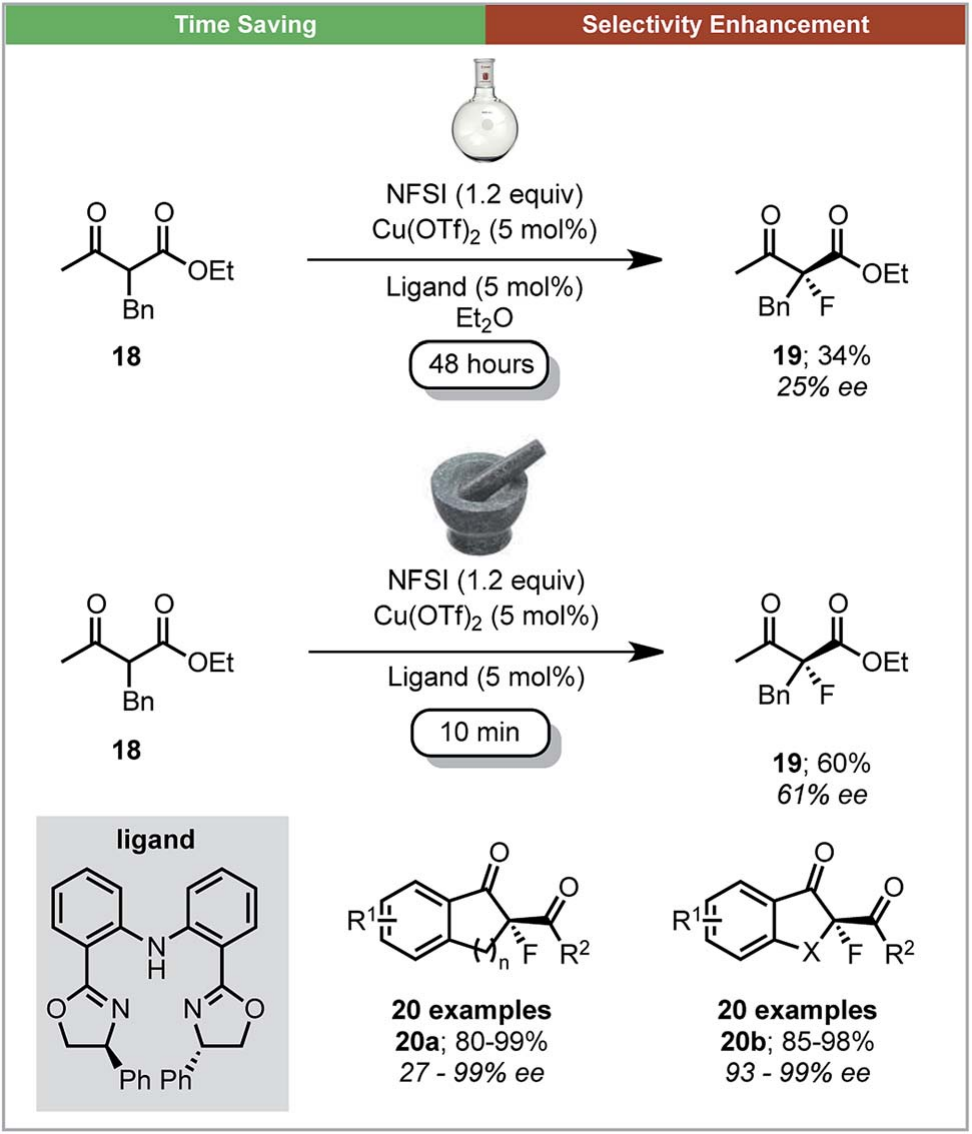
Enantioselective fluorination of β-ketoesters; Xu and co-workers.^54^

Further work on mechanochemical fluorination was performed by Xu and co-workers, who developed a method for the copper catalysed enantioselective fluorination of β-keto esters using *N*-fluorobenzenesulfonimide (NFSI) under ball milling conditions (Scheme 8).^54^ A selection of chiral bis(oxazolines) and bis(azoline) Cu(ii) complexes were generated *in situ* by milling for five minutes before the addition of substrates and NFSI. Using ball-milling, the reaction was complete within four minutes for most substrates, with high enantioselectivities and yields. Using the acyclic β-keto ester **18**, the solution reaction required 48 hours to achieve a yield of 34% and an ee of 24%. This is in stark contrast to the mechanochemical conditions, which give a significantly improved yield (60%) and ee (61%) after only 10 minutes.

The use of organocatalysts negates the need for a metal catalyst, which can lead to greener synthetic processes.^55^ However, there are still limitations such as high catalyst loadings (often 10–20 mol%), limited solvent choice (chlorinated solvents are commonly used), catalyst recovery and long reaction times. In the last decade, ball-milling has been used to improve the performance of organocatalysts under solvent free/LAG conditions, particularly in the area of secondary amine organocatalysis.

**Scheme 9:**
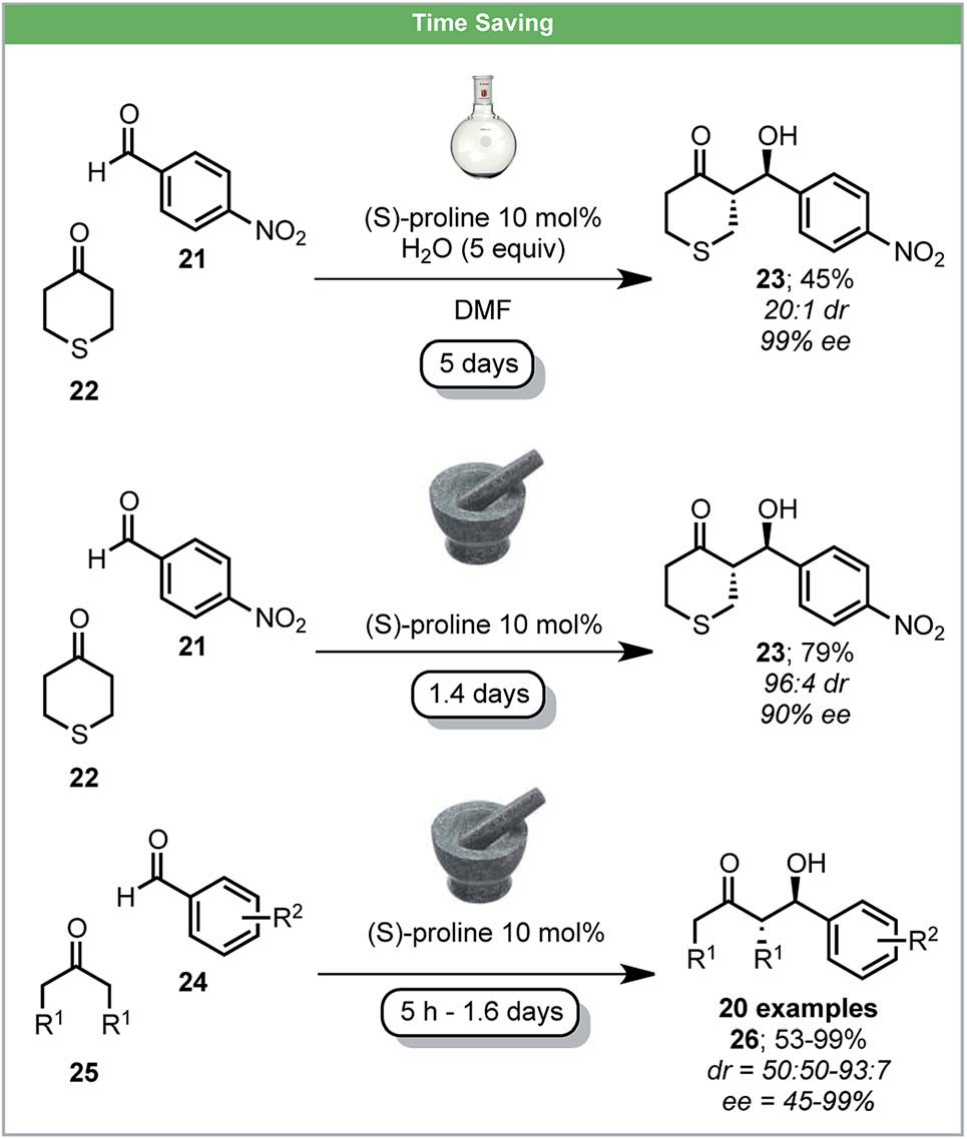
Proline catalysed enantioselective aldol reaction; Bolm and co-workers.^56^

Pioneering investigations of mechanochemical (*S*)-proline-catalyzed asymmetric aldol reactions were carried out by Bolm and co-workers, affording *anti*-aldol products in high yield and with up to 99% ee (Scheme 9).^56^ When comparing the reaction of 4-nitrobenzaldehyde **21** with tetrahydrothiopyran-4-one **22** to analogous solution reactions, a higher yield was achieved with shorter reaction time and similar ee.^57^ Following this pioneering work, several other reports on secondary amine mechanochemical organocatalysis have been published, reporting similar improvements in comparison to solution based reactions.^58^

**Scheme 10:**
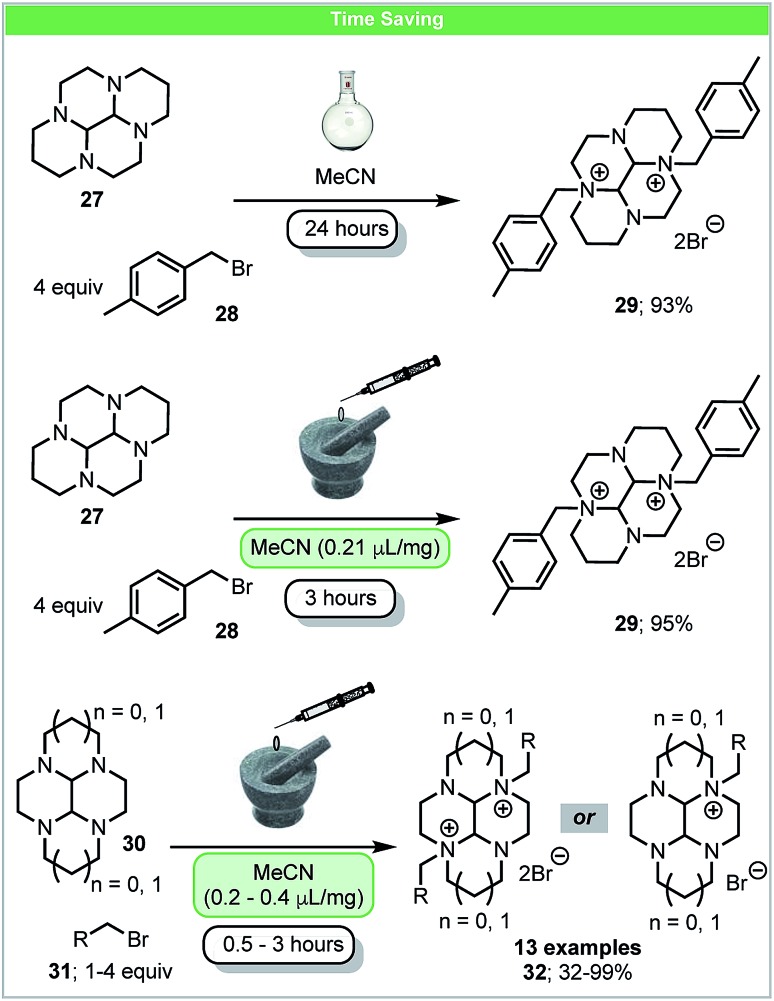
N-Alkylation of bridged cyclam and cyclen derivatives; Archibald and coworkers.^60^

Metal complexes containing macrocyclic polyamine ligands have a range of applications such as medical imaging agents, protein binding agents, antimalarial drugs and catalysis.^59^ In 2016, Archibald and co-workers developed a novel method for N-alkylation of glyoxal-bridged bisaminal derivatives of cyclam (1,4,8,11-tetraazacyclotetradecane) **27** and cyclen (1,4,7,10-tetraazacyclododecane) **28** under mechanochemical LAG conditions (Scheme 10).^60^ Most of the monofunctionalized quaternary ammonium salts could be formed in good to excellent yields by using stoichiometric amounts of alkyl bromides within 30 minutes. Yields of bis-N-alkylated products could be improved by increasing the relative quantity of bromide reagent or the reaction time. Compared to conventional solution methods, using mechanochemistry resulted in a five-fold reduction in the reaction time. It was further suggested that CB-TE2A, widely used to form stable ^64^Cu complexes for PET imaging *in vivo*, could be synthesized within five days using this method. This approach offers a much faster synthetic route compare to the conventional six-step process which takes 35 days developed by Weisman and co-workers.^61^

The examples described above demonstrate that mechanochemical conditions can, in several instances, lead to greatly reduced reaction times compared to the comparable reaction in solution. In some instances this is coupled with an increase in yield. This is an interesting general observation to note, and could be due to running reactions between neat reagents, increased energy input or a contribution from both. Instantaneous localised temperatures in the mill have not been accounted for and could also play a role. Regardless of the explanation, the experimental observation holds true that the use of mechanochemistry can reduce reaction times.

## 3. Selectivity enhancement

As well as reducing reaction times, mechanochemistry has been used to alter or control the selectivity of reaction outcomes. This section describes a few examples of reactions exhibiting a change in selectivity compared to reactions performed in solution.

**Scheme 11:**
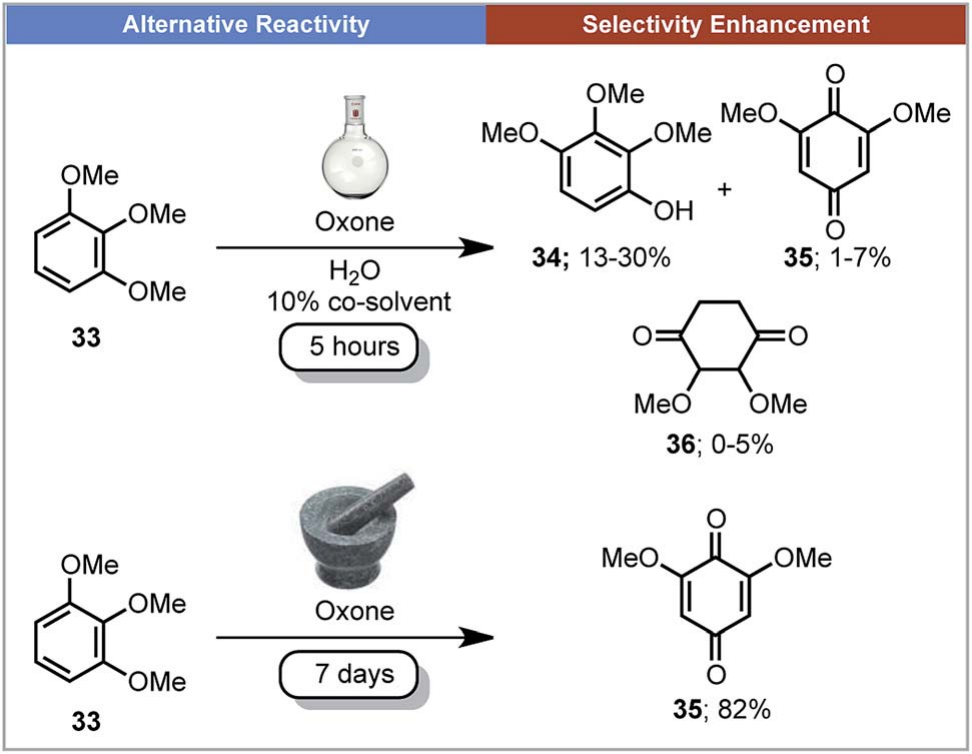
Oxidation of lignin-like methoxylated aromatics; Anastas, Crabtree, Hazari and co-workers.^64^

Plant biomass is a potential feedstock for the production of fuels and chemicals.^62^ The degradation of lignin is one such method to convert the feedstock into commodity chemicals. Mechanochemistry could have the potential to be used in industry for the degradation of lignin, cellulose and chitin.^63^ In 2013, Anastas, Crabtree, Hazari and co-workers reported the mechanochemical oxidation of lignin-like methoxylated aromatic substrates **33** (Scheme 11) using Oxone (potassium peroxymonosulfate) as the oxidant.^64^ When this reaction was carried out in aqueous solution, the major product was 2,3,4-trimethoxyphenol **34**, with several other side-products observed. In contrast, under mechanochemical conditions, quinone **35** was the only product formed. Making use of a rock tumbler/polisher, seven days were required for this transformation.

**Scheme 12:**
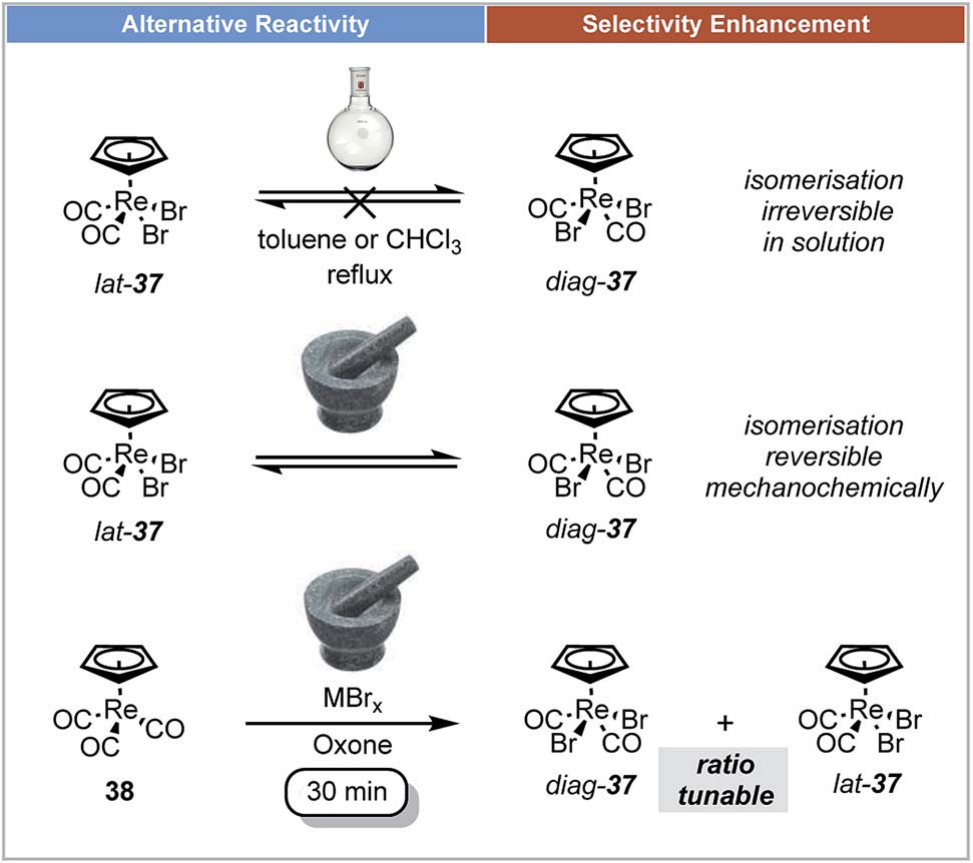
Oxidative halogenation of Re(i) complexes; Friščić and co-workers.^65^

In 2014, Friščić demonstrated that ball milling techniques could offer stereoselective control for the synthesis of organometallic compounds.^65^ The oxidative halogenation of cyclopentadienyl tricarbonyl Re(i) complexes **38** was studied under ball-milling conditions (Scheme 12). The rhenium complexes [CpReX_2_(CO)_2_] **37** and [Cp*ReX_2_(CO)_2_] could be selectively formed in either *diag* (*trans*) or *lat* (*cis*) forms in one step by milling their corresponding cyclopentadienyl tricarbonyl Re(i) complexes with a metal halide (MX_*n*_). It was reported that *diag*-**37** was more thermally stable in the solid state or in solution than its *lat*-**37** form, and that this transformation was irreversible in solution.^66^ It is notable that isomerization of both *diag*-**37** and *lat*-**37** can occur by mechanical treatment instead of thermal treatment. Indeed after 1 h of milling of *diag*-**37** at low temperature it isomerised to a 64 : 36 mixture of *diag*-**37** : *lat*-**37**.

**Scheme 13:**
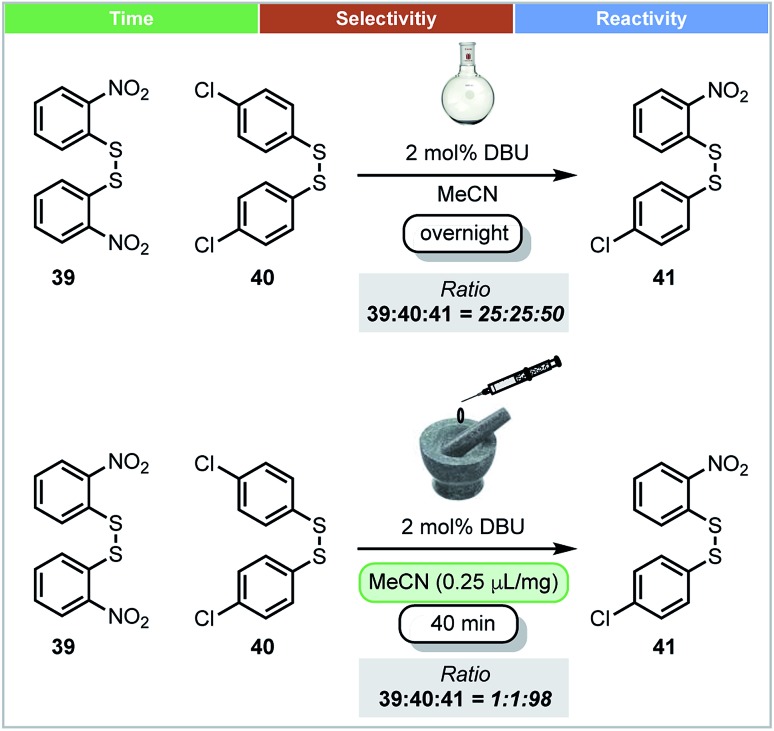
Disulfide metathesis; Friščić and co-workers.^67^

In 2010, Friščić and co-workers demonstrated that thermodynamic equilibrium could be obtained under mechanochemical conditions. Using the base catalysed metathesis of aromatic disulfides as a model reaction (Scheme 13), it was shown that there was a significant difference in the position of equilibrium under mechanochemical and solution-based conditions.^67^ In dilute acetonitrile solution, reactions afforded a ratio of 1 : 1 : 2 between homodimers (**39** and **40**) and heterodimer **41**. However, both LAG (with MeCN) or neat grinding led to almost complete conversion of homodimers, and afforded almost 98% heterodimer **41** (Scheme 13). These different equilibrium compositions could be explained by crystal packing effects, which do not exist in solution, but are a factor for consideration under mechanochemical conditions.

## 4. Different reaction products or different reactivity

Perhaps the most interesting observation arising from running reactions under mechanochemical conditions is that different products can be achieved. This suggests that the kinetics and thermodynamics of some reactions can be significantly altered by using a mill, and leads to the tempting supposition that some molecules are only accessible by this method.

**Scheme 14:**
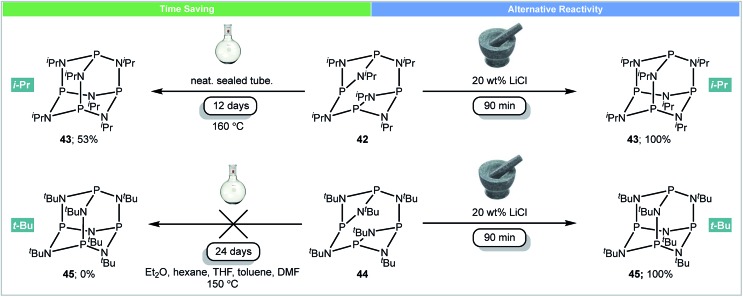
Synthesis of adamantoid cyclophosphazenes; García and co-workers.^68^

In 2016, García and co-workers developed the first example of using mechanochemistry for the synthesis of adamantoid substituted cyclophosphazenes (Scheme 14).^68^ As strong non-carbon covalent backbones, phosphazanes (P–N) are interesting compounds, as they are used as multi-dentate ligands,^69^ catalysts^70^ and antitumor drugs.^71^ It was reported that subjecting the isopropyl-substituted ring [{P(μ-N^i^Pr)_2_}_2_(μ-N^i^Pr)]_2_**42** to mechanochemical milling caused rearrangement to its adamantoid isomer P_4_(N^i^Pr)_6_**43** in 90 minutes.^66^ However, the same rearrangement under high temperature (160 °C) conditions required 12 days (Scheme 14).^72^ The analogous *tert*-butyl-substituted ring [{P(μ-N^*t*^Bu)_2_}_2_(μ-N^*t*^Bu)]_2_**44** could not be converted to its adamantoid isomer P_4_(N^*t*^Bu)_6_**45** using solution methods (reflux in DMF, THF, toluene *etc.*) or prolonged heating.^73^ However, this compound was synthesized for the first time under mechanochemical conditions. Both adamantoid P_4_(N^i^Pr)_6_**43** and P_4_(N^*t*^Bu)_6_**45** could be synthesized in the mixer mill within 90 minutes in the presence of LiCl (20 wt%).

**Scheme 15:**
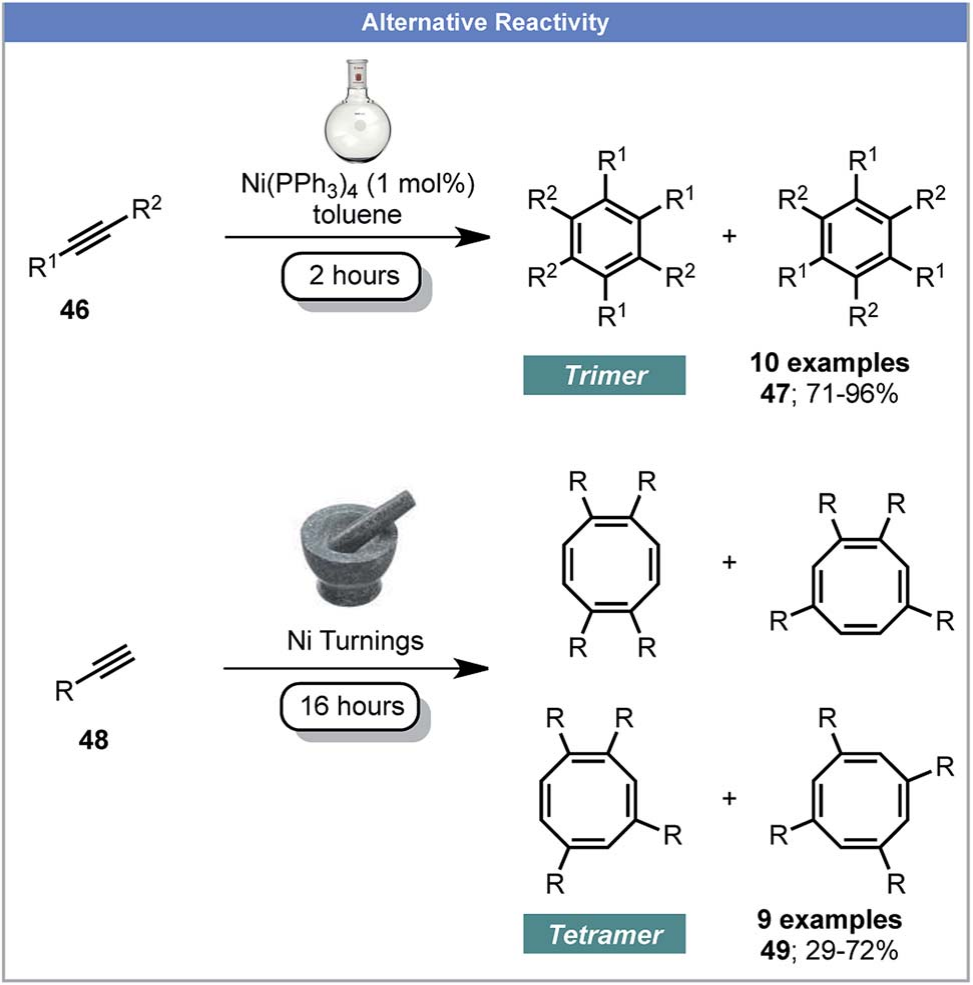
Nickel catalysed cyclotri/tetra-merisation of alkynes; Guan, Mack and co-workers.^75^

It has been shown by Mack and co-workers that using a copper vial or balls instead of conventional milling materials, or adding silver foil enables elemental Cu(0) and Ag(0) to be used as active recyclable catalysts under ball milling conditions.^74^ In 2016, Guan, Mack and co-workers developed a method for the cyclotetramerisation of alkynes to afford cyclooctatetraenes (COT) **49** using recyclable Ni(0) pellets as the catalyst under ball milling conditions (Scheme 15).^75^ Conversely, reactions performed in solution, catalyzed by Ni(0) complexes, yielded the major products as aromatic trimers **47**.^76^ This proof-of-principle study demonstrates the potential of using Ni(0) pellets as active catalyst under mechanochemical conditions, which avoids the use of air-sensitive nickel complexes. It also demonstrates that mechanochemistry may achieve different reaction products compared to conventional solution methods, although grinding with a Ni(PPh_3_)_4_ catalyst would help confirm this.

**Scheme 16:**
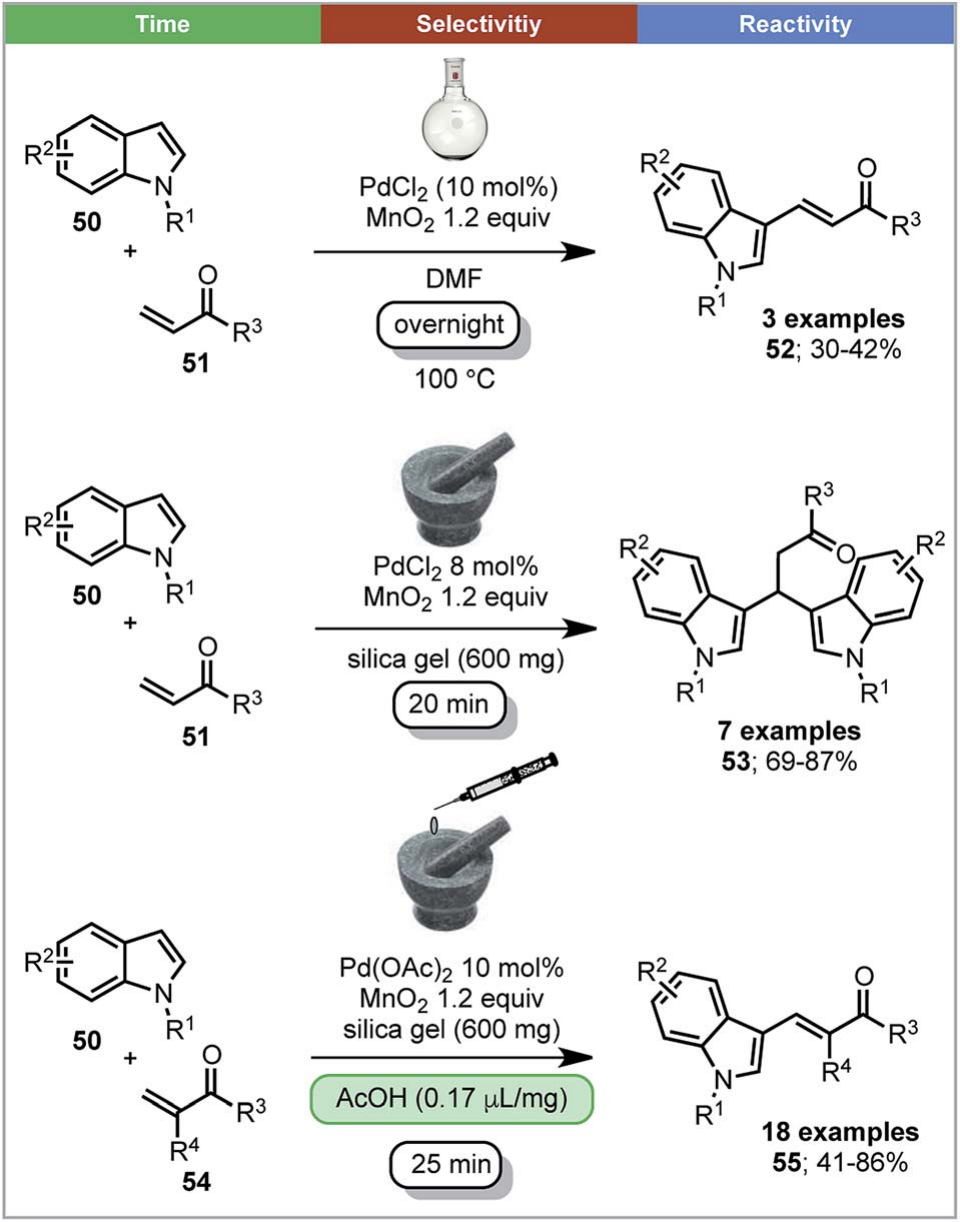
Palladium catalysed oxidative coupling of indoles and acrylates; Su and co-workers.^77^

Su and coworkers reported that 3-vinylindoles **55** and β,β-diindolyl propionates **53** could be synthesized by Pd(ii) catalyzed oxidative coupling reactions between indoles and acrylates with MnO_2_ as oxidant under ball milling conditions (Scheme 16).^77^ It was found that the selectivity of the reaction is influenced by the Pd(ii) source. When Pd(OAc)_2_ was used with acetic acid as additive, high yields of 3-vinylindoles (**55**) were obtained. However, when using PdCl_2_ as a catalyst without a liquid additive, β,β-diindolyl propionates were formed selectively. In contrast, solution conditions; DMF as solvent, 100 °C, overnight, only the 3-substituted-vinylindoles **52** were formed, with no trace of β,β-diindolyl propionates **53** being detected (Scheme 16). A control experiment in solution with added silica and/or acetic acid is not reported. Mechanistic studies using ESI-MS indicated that the formation of a dimeric palladium intermediate under ball milling conditions may be causing the differences in selectivity under solvent based and ball milling conditions.

**Scheme 17:**
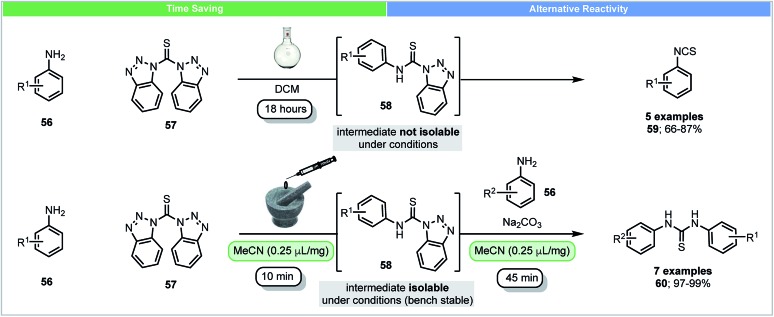
Reaction of anilines with bis(benzotriazolyl)methanethiones and isolation of intermediate; Friščić and co-workers.^79^

In solution, anilines react with bis(benzotriazolyl)methanethiones **57** and form their corresponding isothiocyanates **59** and benzotriazoles through intermediate *N*-(thiocarbamoyl)benzotriazoles **58** (Scheme 17).^78^ Due to the high reactivity of *N*-(thiocarbamoyl)benzotriazoles **58** in solution, they were not isolable using conventional solution chemistry. In 2015, Friščić and co-workers reported the first isolation of *N*-(thiocarbamoyl)benzotriazoles **58** using mechanochemistry.^79^ Excellent yields (>97%) of *N*-(thiocarbamoyl)benzotriazoles could be obtained by milling anilines **56** with bis(benzotriazolyl)methanethiones **57** under LAG conditions within 10 minutes. *N*-(Thiocarbamoyl)benzotriazoles were found to be bench stable in the solid state and could be used for further synthesis of both symmetrical and nonsymmetrical thioureas. These compounds were characterized by solid state magic angle spinning ^13^C NMR spectroscopy. This novel example shows that mechanochemistry could be used to isolate reactive intermediates, which are not isolable in solution. It further demonstrates the ability to form different reaction products, isothiocyanates in solution and thioureas mechanochemically.

### 4.1 Reactivity not possible in solution

**Scheme 18:**
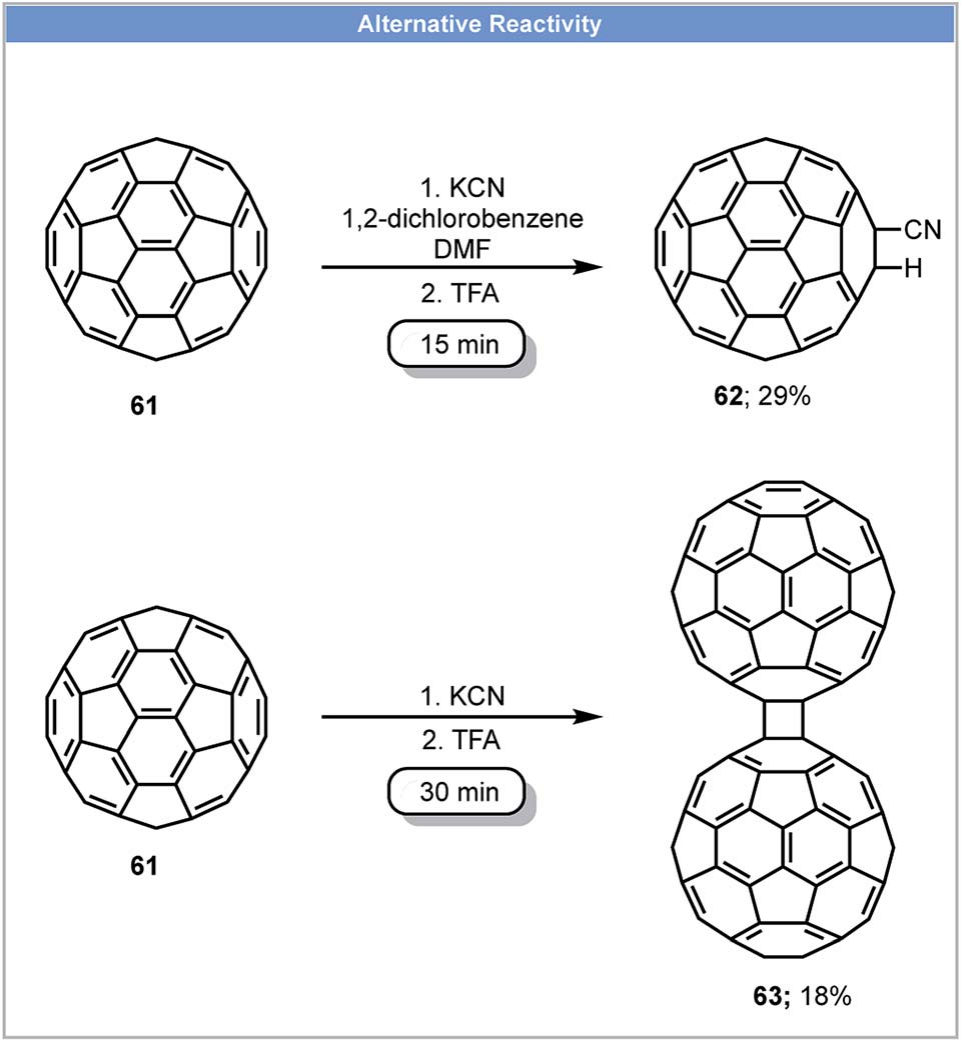
Dimerisation of fullerene; Komatsu and co-workers.^82^

Due to the unique electronic and structural properties of fullerene and its functionalized derivatives, a number of methods for the functionalization of fullerene have been developed.^80^ However, fullerene and fullerene-related materials, such as carbon nanotubes and graphite, often have low solubility in organic solvents and water, which can make their functionalization and application challenging. Mechanochemical techniques offer advantages to reactions of these carbon rich nanostructure materials and have been used to tackle these problems.^81^ In 1997, Komatsu and co-workers developed the first method for the synthesis of a fullerene dimer, C_120_**63** by the use of high speed vibration milling (HSVM) (Scheme 18).^82^ Fullerene dimer C_120_ could be obtained in 30 minutes with 29% yield by milling fullerene C_60_ with KCN at 2800 cycles per minute followed by a trifluoroacetic acid wash. A similar yield could also be achieved by replacing KCN with other reagents such as K_2_CO_3_, KOAc, alkali metals (Li, Na, K) and 4-aminopyridine. A later study revealed that 4% fullerene trimer C_180_ could also be obtained when 4-aminopyridine was used as catalyst.^83^ These compounds could not be obtained using liquid-phase protocols. Under solution based reaction conditions, Wudl and co-workers only obtained cyano functionalised fullerene **62** by stirring fullerene with NaCN in a solvent mixture of 1,2-dichlorobenzene and DMF (Scheme 18).^84^

**Scheme 19:**
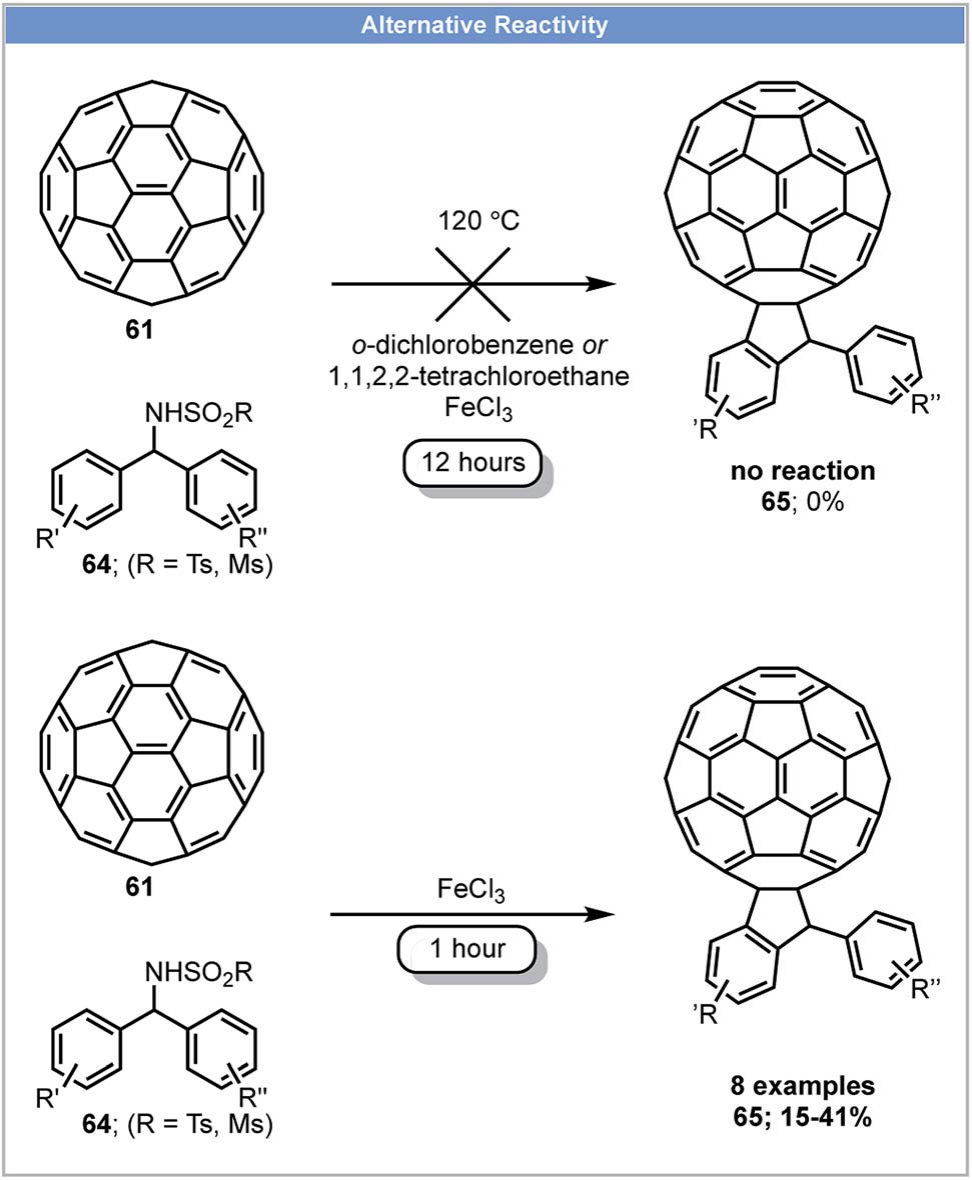
Fullerene functionalisation; Wang and co-workers.^85^

Another example of fullerene functionalisation was reported in 2013 by Wang and co-workers. C_60_-Fused indanes **65** could be synthesised by the reaction of fullerene **61** with *N*-benzhydryl sulphonamides **64** and FeCl_3_ in a ball mill (Scheme 19).^85^ C_60_-Fused indanes (yield: 15–41%) could be obtained within 1 hour under ball milling, whereas no product was formed using solution-based methods (Scheme 19).

**Scheme 20:**
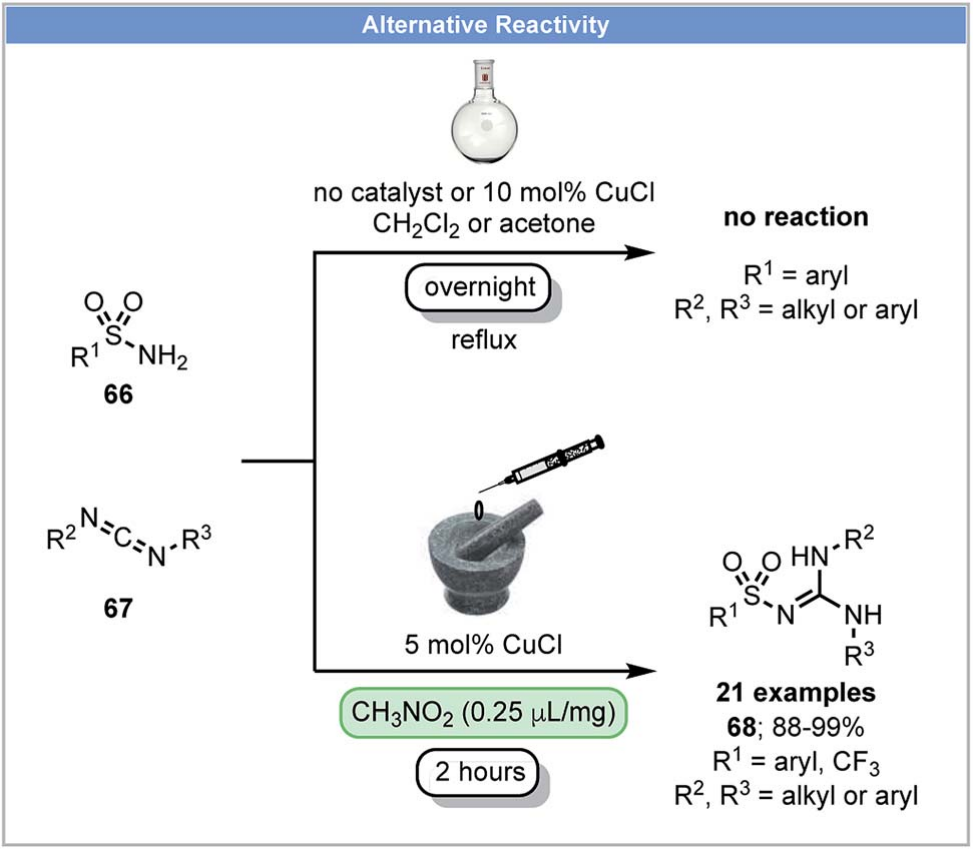
Synthesis of *N*-sulfonylguanidines; Friščić and co-workers.^87^

In 2014, Friščić and co-workers demonstrated that sulfonyl-(thio)ureas could be synthesized from sulfonamides and isocyanates using catalytic CuCl under ball milling conditions.^86^ They subsequently reported that *N*-sulfonylguanidines could be synthesized *via* the copper-catalyzed coupling of arylsulfonamides **66** and carbodiimides **67** under nitromethane LAG conditions (Scheme 20).^87^ Further optimization showed that acetone was the best LAG agent and good yields were obtained after milling for two hours. In contrast, refluxing arylsulfonamides and carbodiimides in solvents (DCM or acetone) overnight with or without CuCl led to no product formation.

**Scheme 21:**
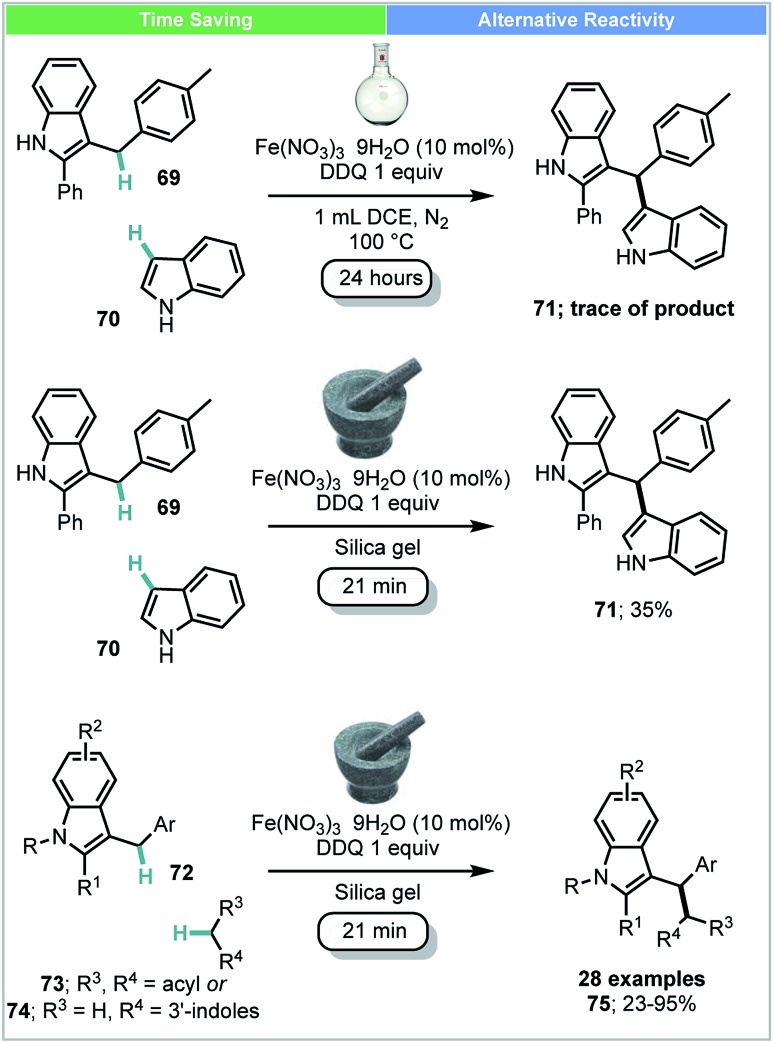
Iron catalysed cross dehydrogenative coupling; Su and co-workers.^88^

Su and co-workers developed an Fe(iii)-catalyzed cross-dehydrogenative coupling (CDC) of 3-benzylic indoles **72** with compounds bearing acidic methylene groups (**73** & **74**) under ball-milling conditions (Scheme 21).^88^ This catalytic system was also successfully used for the synthesis of bisindoles **71** (yield: 24–77%). This was in contrast to the comparable solution-based reaction carried out at 100 °C under N_2_ gas atmosphere using DCE as solvent (Scheme 21), under which conditions, only trace amounts of product were observed.

Synthesising organometallics under mechanochemical conditions can extend the scope of suitable reactants without concern for solubility or potential interference from solvent, such as through coordination or quenching.

**Scheme 22:**
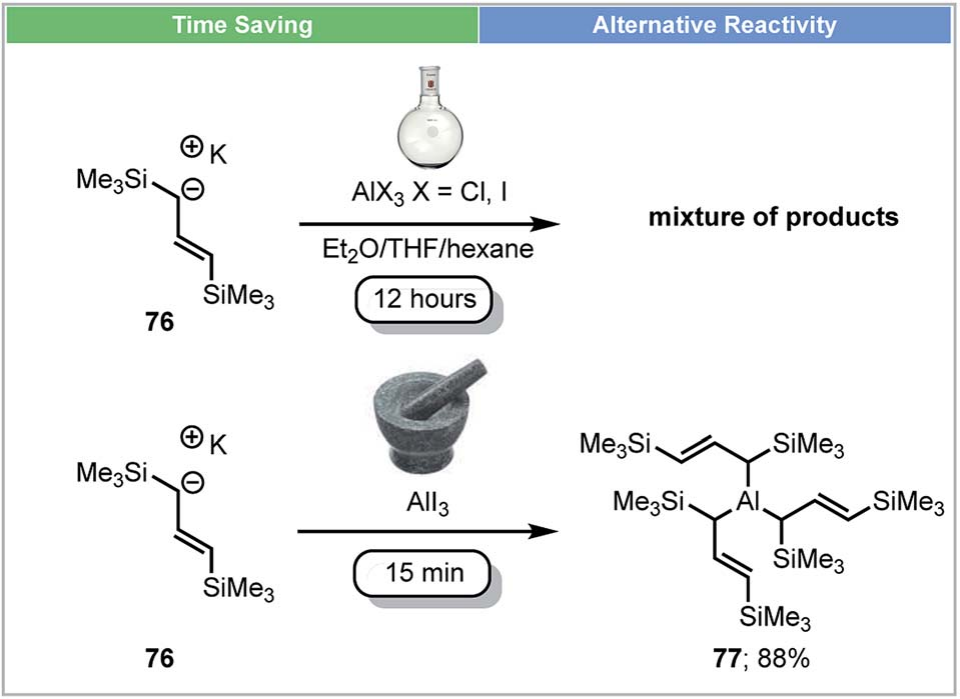
Synthesis of unsolvated trisallylaluminium complex; Hanusa and co-workers.^89^

In 2014, an unsolvated tris(allyl)aluminium complex **77** was first isolated and reported by Hanusa and co-workers using mechanochemical conditions (Scheme 22).^89^ Attempts to synthesise this complex by stirring K[1,3-(SiMe_3_)_2_C_3_H_3_] **76** with AlX_3_ (X = Cl, I) in different solvents (Et_2_O, THF, hexane) led to a mixture of unidentified products. The unsolvated tris(allyl)aluminium complex **77** (yield up to 88%) could be obtained within five minutes using a planetary ball mill. The reactivity of this newly synthesized complex **77** was tested by reaction with benzophenone in hexane at –78 °C. Compared to the THF adduct (C_3_H_5_)_3_Al(THF), this unsolvated tris(allyl)aluminium complex **77** showed a faster reaction rate, despite its bulkier ligand.

**Scheme 23:**
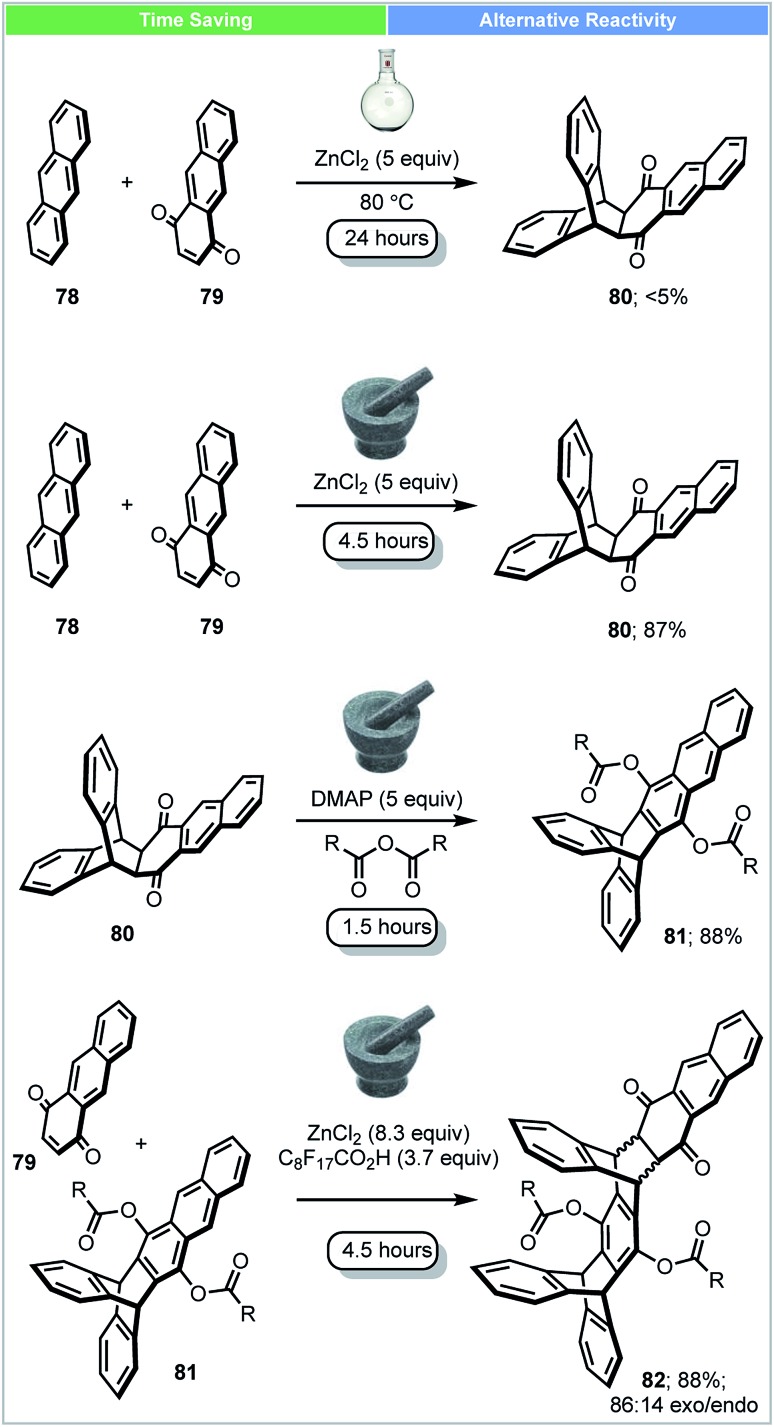
Synthesis of extended iptycenes; Swager and co-workers.^91^

Iptycenes have three dimensional rigid molecular architectures, and potentially have multiple applications, such as molecular machines, novel liquid crystals and porous polymers.^90^ In 2016, Swager and co-workers showed that highly functionalized iptycenes (molecular weight > 2000 g mol^–1^) could be synthesized by iterative Diels–Alder/aromatisation reactions under solvent-free conditions (Scheme 23).^91^ A good yield (87%) of adduct **80** could be achieved in a ball mill using ZnCl_2_ as a Lewis acid. In contrast, only 5% of this material was obtained when the reaction was carried out in solution in the presence of ZnCl_2_ at 80 °C after 24 h. To achieve extended iptycenes **82**, perfluorononanoic acid (C_8_F_17_COOH) was used as an additive to increase the acidity and catalytic performance of the milled reaction. This multistep solvent free synthesis demonstrates the strengths of mechanochemical methods for the synthesis of large functionalized extended iptycenes over traditional methods.

## 5. Conclusions and outlook

We hope that this overview will provide inspiration for others to explore the relatively uncharted territory of mechanochemistry for organic synthesis. Mechanochemistry *is* still in the early phase of its development, especially as a technique for organic synthesis. However, it has already been shown that a wide variety of important transformations are possible in the absence of solvent. Furthermore, it has been demonstrated that it can be scaled to manufacture levels. Not only driven by the inherent appeal from a sustainability perspective, we have highlighted some examples that are particularly interesting when compared to reactions in solution. Notably, we have shown that there exist a number of examples where using mechanochemistry enables faster, more selective and novel reactivity, much of which is not yet predictable or expected, but is certainly exciting, intriguing and sometimes perplexing! It is currently standard practice during reaction optimisation to screen solvents. However, as shown here, a lack of solvent could well lead to improved or completely unexpected results.

Therefore, we are optimistic that there remains a large part of unexplored ‘chemical space’ and fundamental ‘chemical understanding’ hidden within this technique. With much experimentation, more will be discovered about what is possible mechanochemically and the technique will build into one which is predictable. For now, why don't you have a go?^92^

## Conflicts of interest

There are no conflicts to declare.
